# Methylenedioxypyrovalerone (MDPV) mimics cocaine in its physiological and behavioral effects but induces distinct changes in NAc glucose

**DOI:** 10.3389/fnins.2015.00324

**Published:** 2015-09-17

**Authors:** Ken T. Wakabayashi, Suelynn E. Ren, Eugene A. Kiyatkin

**Affiliations:** In-Vivo Electrophysiology Unit, Behavioral Neuroscience Branch, National Institute on Drug Abuse – Intramural Research Program, Department of Health and Human Services, National Institutes of HealthBaltimore, MD, USA

**Keywords:** electrochemistry and electrochemical processes, brain thermorecording, extracellular glucose concentration, vascular tone, metabolism

## Abstract

Methylenedioxypyrovalerone (MDPV) is generally considered to be a more potent cocaine-like psychostimulant, as it shares a similar pharmacological profile with cocaine and induces similar physiological and locomotor responses. Recently, we showed that intravenous cocaine induces rapid rise in nucleus accumbens (NAc) glucose and established its relation to neural activation triggered by the peripheral drug actions. This study was conducted to find out whether MDPV, at a behaviorally equivalent dose, shares a similar pattern of NAc glucose dynamics. Using enzyme-based glucose sensors coupled with amperometery in freely moving rats, we found that MDPV tonically decreases NAc glucose levels, a response that is opposite to what we previously observed with cocaine. By analyzing Skin-Muscle temperature differentials, a valid measure of skin vascular tone, we found that MDPV induces vasoconstriction; a similar effect at the level of cerebral vessels could be responsible for the MDPV-induced decrease in NAc glucose. While cocaine also induced comparable, if not slightly stronger peripheral vasoconstriction, this effect was overpowered by local neural activity-induced vasodilation, resulting in rapid surge in NAc glucose. These results imply that cocaine-users may be more susceptible to addiction than MDPV-users due to the presence of an interoceptive signal (i.e., sensory cue), which may result in earlier and more direct reward detection. Additionally, while health complications arising from acute cocaine use are typically cardiovascular related, MDPV may be more dangerous to the brain due to uncompensated cerebral vasoconstriction.

## Introduction

Methylenedioxypyrovalerone (MDPV) is a new psychoactive drug of abuse that has recently gained popularity with the emergence of “bath salts” (Baumann et al., 2013; Iversen et al., 2014; Watterson et al., 2014). MDPV is often considered a cocaine-like drug as its primary effects are mediated via an interaction with the same monoamine transporters as cocaine, resulting in similar physiological and locomotor effects (Ross et al., 2012; Baumann et al., 2013; Gregg and Rawls, 2014). However, while cocaine has relatively equal affinities with all the monoamine transporters, MDPV shows differing affinities. Specifically, MDPV is exceptionally potent at the dopamine (DA) transporter and shows a lower affinity at the norepinephrine transporter, while being virtually ineffective at the serotonin transporter (Baumann et al., 2013). These differences may account for the higher potency of MDPV compared to cocaine (~10x) as well as the distinct behavioral, physiological, and psycho-emotional effects of these drugs (Fantegrossi et al., 2013; Watterson et al., 2014; Aarde et al., 2015). Although humans typically orally consume MDPV, rats intravenously (iv) self-administer this drug at rates comparable to cocaine (Aarde et al., 2013, 2015; Bonano et al., 2014; Watterson et al., 2014), indicating a high addictive potential and, in light of MDPV's low cost and much higher potency, a high probability for significant health complications during drug overdose.

Recently, we showed that iv cocaine induces biphasic increases in nucleus accumbens (NAc) glucose levels, characterized by a first, rapid phase that remains stable following repeated drug injections and a second tonic phase that diminishes following repeated injections (Wakabayashi and Kiyatkin, 2015a). Although it is known that cocaine induces peripheral vasoconstriction (Knuepfer and Branch, 1992; Brown and Kiyatkin, 2005), a similar effect on the cerebral vessels should result in a decrease, rather than a rapid surge in glucose. Therefore, we hypothesized that cocaine-induced systemic vasoconstriction is superseded by drug-induced local neuronal activity-dependent vasodilation, triggered by rapid, transient excitation of accumbal neurons (Kiyatkin and Brown, 2007). Although these rapid central effects occurring within the injection are often attributed to the direct actions of cocaine in the brain, they are in fact mediated via a peripherally triggered mechanism. Previously, we showed that cocaine-methiodide, a cocaine analog that cannot cross the blood-brain barrier, induces many of the same ultra-fast responses as cocaine itself, including skin vasoconstriction (Brown and Kiyatkin, 2005), cortical EEG desynchronization (Kiyatkin and Smirnov, 2010), excitation of accumbal neurons (Kiyatkin and Brown, 2007), and a rapid rise in NAc glucose (Wakabayashi and Kiyatkin, 2015a). Therefore, by stimulating peripheral sensory afferents, iv cocaine induces a neural signal that is rapidly transmitted to the CNS, resulting in neural activation, local neural activity-dependent vasodilation, and enhanced entry of glucose and oxygen into the brain's extracellular space. Due to this peripheral action, cocaine administration produces its own sensory cue which may result in earlier and more direct reward signaling, that could be important for the acquisition of drug-taking behaviors (see Wise and Kiyatkin, 2011 for review).

MDPV, like cocaine, induces metabolic brain activation and peripheral vasoconstriction (Kiyatkin et al., 2015), two determinants of extracellular glucose levels (Kiyatkin et al., 2013). As there is very limited knowledge regarding the neurochemical and metabolic effects of MDPV, this study was designed to evaluate whether MDPV shares a similar pattern of NAc glucose dynamics with cocaine. Such data could provide valuable information not only on possible contributions of the peripheral and central actions of this drug on metabolic neural responses, but also may have implications regarding the addiction liability of MDPV. Here, enzyme-based glucose sensors coupled with constant-potential amperometery were used in freely moving rats to examine NAc glucose changes induced by MDPV administered iv at a dose optimal for drug self-administration (0.1 mg/kg). These changes were compared to those induced by iv cocaine injected at an optimal self-administering dose (1 mg/kg). Cocaine-induced changes in NAc glucose were described in detail previously (Wakabayashi and Kiyatkin, 2015a); here they were re-analyzed and shown to contrast them to changes induced by MDPV at a behaviorally equipotential dose. To mimic human conditions where these drugs are repeatedly used in a relatively short time frame, we examined the effects of four repeated injections of MDPV and cocaine during 1 day-long recording session.

Our electrochemical recordings were supplemented by direct monitoring of temperatures in the NAc, temporal muscle and skin and locomotor activity. This three-point thermorecording paradigm allowed us to compare the effects of each drug on intra-brain heat accumulation due to metabolic activation and peripheral vascular tone (Kiyatkin, 2010). Using this two-level analysis, we were able to parse the mechanisms underlying distinct patterns in NAc glucose induced by MDPV and cocaine, as well as the within-session changes in these patterns following repeated drug administration.

## Materials and methods

### Animals and housing

Twenty-two male Long-Evans rats (440 ± 20 g at the time of surgery) supplied by Charles River Laboratories (Greensboro, NC) were housed individually in a temperature-, humidity-, and light-controlled room (12/12 h light/dark cycle, lights on at 07:00) with free access to food and water. Protocols were performed in compliance with the Guide for the Care and Use of Laboratory Animals (NIH, Publication 865-23) and were approved by the NIDA-IRP Animal Care and Use Committee.

### General structure of the study

This study combines the results of two experiments. In *Experiment I* (*n* = 11 rats), we examined the changes in NAc extracellular glucose levels induced by iv injections of MDPV in freely moving rats during a single, day-long session. For comparison, we analyzed these results with respect to changes induced by iv cocaine administered using an identical experimental protocol (see Wakabayashi and Kiyatkin, 2015a). Rats were surgically prepared for electrochemical recordings (see below) that were conducted during one daily session. In *Experiment II* (*n* = 8 rats), using the same in-session protocol as in Experiment I, we examined the effects of MDPV or cocaine on NAc, temporal muscle, and skin temperatures. Rats for this experiment were surgically implanted with chronic thermocouple sensors. In a counterbalanced design, MDPV and cocaine were administered to well-habituated rats during two interleaved sessions such that each rat received both drugs. The use of both within- and between-animal comparisons allowed us to minimize the number of rats with chronic implants and increase statistical strength of our findings.

#### Common procedures in both experiments

In both experiments, rats were implanted with a chronic jugular catheter, which ran subcutaneously to a head mount constructed specifically for either experiment (see below), made from dental acrylic and secured to the skull by three stainless steel bone screws. Rats were allowed a minimum of 4 days of post-operative recovery; jugular catheters were flushed daily with 0.2 ml heparinized saline (10 units/ml) to maintain patency. At the onset of both experiments, the injection port of the jugular catheter on the head mount was connected to plastic catheter extensions that allowed stress- and cue-free delivery of saline and the tested substance from outside the cage, thus minimizing possible detection of the iv drug injection by the rat.

All rats underwent similar habituation to the testing environment (a minimum of 6 h a day for 3 consecutive days) prior to testing. Similarly, cages for both experiments were equipped with four infrared motion detectors (Med Associates) to measure locomotor activity. On test day, experiments always began 5 days after surgery and were conducted during the light-phase of the cycle (10:00–17:00).

### Experiment I

#### Surgical preparations

Each rat used was surgically prepared for electrochemical recordings as described in detail previously (Kiyatkin and Lenoir, 2012; Wakabayashi and Kiyatkin, 2014, 2015a,b). Briefly, under general anesthesia (Equithesin 0.33 ml/100 g, ip), rats were implanted with a BASi cannula (Bioanalytical Systems, Inc.; West Lafayette, IN) for future insertions of a biosensor in the medial sector of the nucleus accumbens (NAc shell). The target coordinates were: AP +1.2 mm, ML ±0.8 mm, and DV 7.3 mm, according to the stereotaxic atlas of Paxinos and Watson (Paxinos and Watson, 1997). The guide cannula hub was fixed to the skull with a head mount constructed from dental acrylic that was secured using three stainless steel bone screws. During the same surgical procedure, rats were also implanted with a chronic jugular catheter, as described above.

#### Fixed-potential amperometry with enzyme-based glucose sensors

Commercially produced glucose oxidase-based biosensors (Pinnacle Technology, Inc.) coupled with fixed-potential amperometry have been extensively used in our previous studies (Kiyatkin and Lenoir, 2012; Kiyatkin et al., 2013; Kiyatkin and Wakabayashi, 2015; Wakabayashi and Kiyatkin, 2015a,b). These reports describe in detail multiple issues regarding the sensitivity/selectivity of these sensors, their *in vitro* and *in vivo* performance, and possible physical and chemical contributions that could be evaluated and controlled for providing high reliability and accuracy of electrochemical measurements of extracellular glucose fluctuations.

Briefly, glucose sensors are prepared from Pt-Ir wire of 180 μm diameter, with a sensing cavity of ~1 mm length on its tip. The active electrode is incorporated with an integrated Ag/AgCl reference electrode. On the active surface, glucose oxidase converts glucose to glucono-1,5-lactone and hydrogen peroxide (H_2_O_2_), which is detected as an amperometric oxidation current generated by a +0.6 V applied potential (Hu and Wilson, 1997). The potential contribution of ascorbic acid to the measured current is competitively reduced by co-localizing ascorbic acid oxidase enzymes on the active surface of the sensor. This enzyme converts ascorbic acid to non-electroactive dehydroascorbate and water. In addition, a negatively charged Nafion polymer layer under the enzyme layer serves to exclude endogenous anionic compounds (Hu and Wilson, 1997).

Glucose sensors were calibrated immediately before and after each *in vivo* experiment. *In vitro* calibrations were conducted in PBS (pH 7.4) by incrementally increasing the concentration of glucose (Sigma-Aldrich) from 0 to 0.5, 1.0, and 1.5 mM followed by a single addition of ascorbate (25 μM). Within this physiological range of glucose levels (Fellows and Boutelle, 1993; McNay et al., 2001), glucose sensors used in this study produced incremental linear current increases. Mean sensitivity to glucose was 2.82 ± 0.22 nA/0.5 nM at 22–23°C and 5.51 nA at 37°C. Glucose sensors showed low sensitivity to ascorbate (0.06 ± 0.04 nA/25 μM at 22–23°C) and, as shown previously, they were only minimally sensitive to dopamine at its physiological levels (5–50 pA/10–100 nM). Glucose sensors remained equally sensitive to glucose and selective against ascorbate during post-recording *in vitro* calibrations (2.57 ± 0.16 nA/0.5 mM and 0.24 nA ± 0.12/25 μM, respectively). Since our previous *in vitro* and *in vivo* tests revealed the importance of control recordings with glucose-null sensors for reliable detection of glucose (Kiyatkin and Wakabayashi, 2015), in addition to experiments with a glucose-active sensors (*n* = 8), similar experiments with glucose-null sensors were conducted on another group of rats (*n* = 4).

#### Protocol

In the electrochemical experiment (*n* = 11 rats), we examined changes in NAc extracellular glucose levels ([glucose]) induced by iv injections of MDPV. Electrochemical recordings occurred in an electrically insulated chamber (38 × 47 × 47 cm) under continuous dim illumination (20 W red light bulb), with a room wide air filter fan providing background noise.

At the beginning of each experimental session, rats were minimally anesthetized (<2 min) with isoflurane and a calibrated glucose sensor was inserted into the brain through the guide cannula. The sensor was connected to the potentiostat (Model 3104, Pinnacle Technology) via an electrically shielded flexible cable and a multi-channel electrical swivel. Testing began ~140 min after insertion of the sensor when the baseline currents relatively stabilized and continued for 3–5 h.

Experiments with MDPV and cocaine had identical protocols that differed only by the drug. In both experiments, we used null sensors. Each rat received one saline injection (0.2 ml delivered over 20 s) and four injections of either MDPV (0.1 mg/kg dissolved in 0.2 ml saline and delivered over 20 s) or cocaine (1 mg/kg dissolved in 0.2 ml saline and delivered over 20 s). Intervals between drug injections were 60 min for both drugs. This time interval greatly exceeds the half-life of iv cocaine (Tsibulsky and Norman, 1999) and is sufficient for restoring both physiological and behavioral effects of cocaine at the low dose used in this study (Brown and Kiyatkin, 2005). At the end of each session, rats were iv injected with Equithesin (0.8 ml delivered over 2 min) to induce general anesthesia. Then, the rat was disconnected from the potentiostat and the sensor was removed for post-recording calibrations. Rats were then transcardially perfused with room-temperature PBS (pH 7.4) followed by 10% formalin. Brains were later sectioned on a cryostat to a thickness of 30 μm. The location of the sensors was verified using the stereotaxic atlas of Paxinos and Watson (Paxinos and Watson, 1997).

#### Electrochemical data analyses

Electrochemical data were sampled at 1 Hz (i.e., mean current over 1 s) using the PAL software (Version 1.5.0, Pinnacle Technologies) and analyzed using two time resolutions. Slow changes in electrochemical currents were analyzed with 1-min quantification bins using an analysis window of 5 min before and 60 min after each iv injection. Rapid current changes were analyzed with 8-s bins for 30 s before and 720 s after the onset of iv injections of MDPV and cocaine, respectively. Since the baseline currents slightly varied in amplitude between individual glucose electrodes, absolute current changes were transformed into relative changes taking a basal value before each event (30 s for slow and 8 s for rapid time-course analyses) as 0 nA. These current changes were then transformed into glucose concentration (μM) based on sensor sensitivity determined during pre-recording *in vitro* calibrations and adjusted by the temperature coefficient (95.6%) determined in previous analytical studies (Kiyatkin et al., 2013). The data were also presented as a percent change based on previously determined basal levels of glucose in the NAc (range of 540–700 μM; (Kiyatkin and Lenoir, 2012; Wakabayashi and Kiyatkin, 2015a; Wakabayashi et al., 2015). These values are close to previous electrochemical and microdialysis estimates conducted in awake animals (0.4–0.7 mM; (Fellows et al., 1992; Lowry et al., 1994; McNay and Gold, 1999), but lower than data obtained in anesthetized rats (Silver and Erecinska, 1998).

### Experiment II

#### Surgical preparations

In Experiment II, rats underwent the three-point thermocouple electrode implantation procedure described in detail elsewhere (Kiyatkin et al., 2015). Briefly, under general anesthesia, we implanted miniature copper-constantan thermocouple probes (125 μm in diameter) in the nucleus accumbens (NAc) shell (AP +1.2 mm; L 0.9 mm; DV 7.2–7.4 mm), deep temporal muscle, and subcutaneously along the nasal ridge with the tip approximately 15 mm anterior to bregma. Testing cages were light- and sound-attenuated and equipped with an electrical swivel and a flexible cable that attached to the implanted thermoelectrodes during recording sessions. Temperature data were continuously recorded at a 10-s resolution using Thermes-16 (Physitemp Instruments).

#### Protocol

The experimental protocol was identical to that in Experiment I, except for one difference: each rat was exposed to both MDPV and cocaine in two separate sessions. These sessions were strictly counterbalanced (four with MDPV as a first drug and four with cocaine as a first drug) and separated by 1 free day. Iv catheters in this experiment were flushed three times with saline between recording sessions to prevent contamination of the injected solutions.

#### Temperature data analyses

Temperature responses were analyzed as absolute changes, as changes relative to the immediate pre-injection baseline values, and as NAc-Muscle and Skin-Muscle temperature differentials (i.e., the difference between relative temperature changes in the corresponding locations) and presented as mean values ± standard error. Slow changes in both temperature and locomotion were analyzed in 1-min quantification bins using an analysis window of 10-min before and 60-min after each iv drug injection. Rapid changes were analyzed in 10-s bins for 120-s before and 900 s after each iv drug injection.

Since the brain and temporal muscle receive arterial blood from the same common carotid artery and are equally exposed to blood-delivered heat from the body, NAc-Muscle temperature differentials show the source of heat production, providing a measure of drug-induced metabolic brain activation. Skin temperature is determined by the state of peripheral vessels, but also depends on the temperature of arterial blood inflow. Therefore, Skin-Muscle temperature differentials exclude the latter contribution and serves as an accurate measure of peripheral vascular tone (Kiyatkin, 2010).

#### Statistical analyses

Statistical data analyses included the use of One-Way repeated measure (RM) ANOVAs to find time periods where there was a significant post-injection main effect and individual bins were compared with respect to baseline using a Fisher *post-hoc* test. The latency of the glucose response was determined based on the first data point significantly different from baseline (*p* < 0.05). Two-Way ANOVAs and other appropriate tests were used to compare the effects of MDPV and cocaine. We used area under the curve (AUC) for the period of drug effect to fully capture the combined, overall effect of MDPV and cocaine, as well as each response following repeated, in-session drug injections. To examine the relationships between individual temperature and neurochemical parameters we also used correlation and regression analyses. For clarity, major quantitative results of statistical comparisons are presented in figure captions.

## Results

### General pattern of fluctuations in NAc glucose induced by MDPV: comparison with cocaine

**Figure 1:**
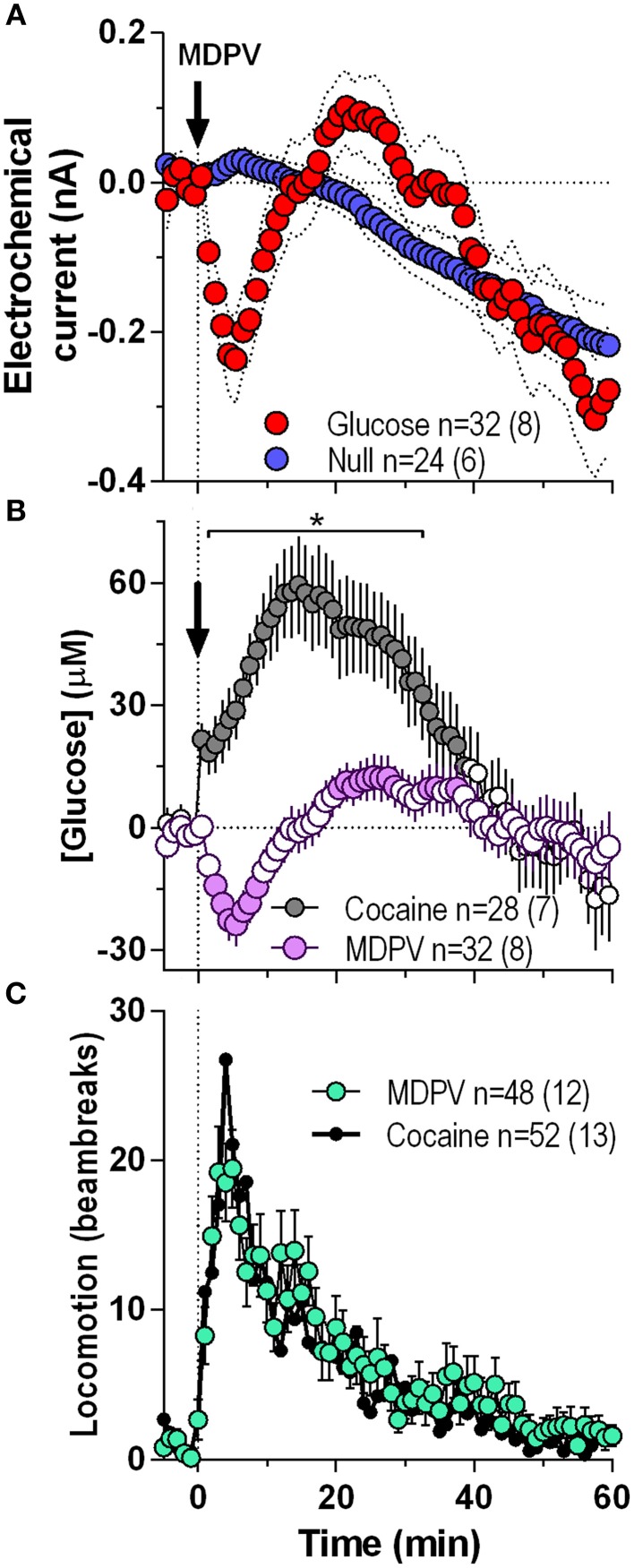
**Overall changes in NAc [glucose] induced by intravenous MDPV and cocaine**. **(A)**, the mean (±SEM) change in currents recorded by glucose (red) and glucose-null (blue) sensors shown in 1-min bins from 5 min before to 60 min after MDPV injections. **(B)**, the mean ± SEM changes in NAc [glucose] induced by MDPV and cocaine. **(C)**, the overall locomotor response to MDPV (green) and cocaine (black line). n, the number of drug injections and number of rats averaged for this analyses. Filled symbols in **(B)** show values significantly different from baseline (*p* < 0.05; Fisher *post-hoc* test after significant drug effect detected by One-Way RM ANOVA). Asterisk in **(B)** denotes the interval where there were significant differences between drugs detected by a Fisher *post-hoc* test after significant main effects of drug and Drug x Time interactions were found by Two-Way RM ANOVA.

Initially we combined all MDPV-induced glucose responses independent of their injection number within the session and analyzed them at a 1-min resolution for 60 min post-injection (Figure 1). In contrast to a weak, not-significant increase followed by a slow decrease below baseline detected by glucose-null sensors (blue line), currents detected by the glucose sensors (red line) rapidly decreased after the MDPV injection, but began to rebound from ~5 min post-injection exceeding the values detected by null sensor (Figure 1A). By subtracting values detected by glucose and glucose-null sensors, we found that glucose levels modestly but significantly decreased (~25 μM, nadir ~4 min) and then rebounded to levels slightly higher than the pre-injection baseline from ~20 min post-injection [*F*_(31, 1740)_ = 5.51, *p* < 0.001; Figure 1B]. These changes contrasted to those induced by cocaine, which elicited a strong, bimodal increase in glucose (~60 μM, peak ~14 min) for ~45 min post-injection [*F*_(27, 1620)_ = 15.35, *p* < 0.001; Figure 1B). As such, MDPV and cocaine induce opposite changes in NAc glucose [Two-Way ANOVA: main effect *F*_(1, 56)_ = 7.21, Drug × Time interaction *F*_(60, 3360)_ = 10.73; both *p* < 0.001], which differ in both the latency and duration. However, both MDPV and cocaine similarly increased locomotion for ~40 min post-injection, with no between-drug differences within the entire analysis interval (Figure 1C).

### NAc glucose responses following repeated injections of MDPV: comparison with cocaine

**Figure 2:**
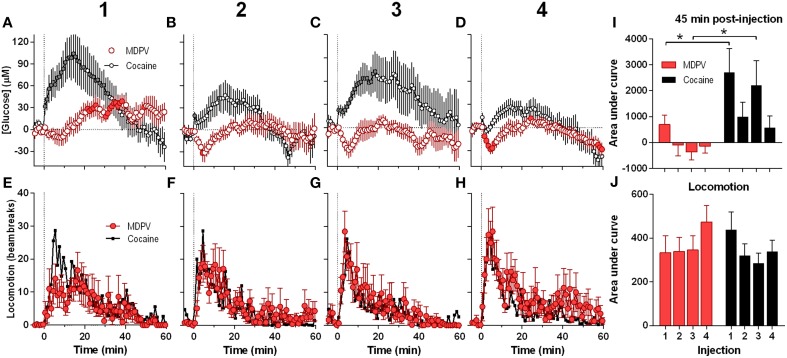
**Relative changes in NAc [glucose] and locomotion induced by MDPV and cocaine injections (1–4) assessed at low temporal resolution (1-min bins)**. **(A–D)**, The mean (±SEM) change in NAc [glucose] (μM) induced by MDPV (red circles) and cocaine (gray circles) for 60 min post-injection. **(E–H)**, changes in locomotor activity (mean ± SEM; counts/min). Vertical hatched lines (at 0 min) marked the onset of 20-s injections of MDPV and cocaine. Horizontal dotted lines show basal levels (=0 μM). Concentration changes for the 60 min analysis window were significant for each MDPV [One-Way RM ANOVA, *F*_(7, 420)_ = 2.63, 1.78, 1.48, and 3.54, all *p* < 0.05, respectively] and cocaine injection [*F*_(6, 360)_ = 7.07, 3.63, 2.30, and 5.61]. Individual values significantly different from baseline (*p* < 0.05 Fisher test) are shown as filled symbols. MDPV induced significant locomotor activation after each injection [*F*_(11, 671)_ = 2.06, 3.05, 3.80, 2.59 for injections 1–4, respectively; *p* < 0.05, for clarity *post-hoc* test results not shown]. Right panels **(I,J)** show mean ± SEM values of glucose and locomotor responses induced by MDPV and cocaine as assessed by the area under the curve for 45 min post-injection. Two-Way RM ANOVA analysis revealed a main effect of drug [*F*_(1, 39)_ = 11.09, *p* < 0.05] and Injection [*F*_(3, 39)_ = 3.28, *p* < 0.05] on NAc glucose. Interaction was not significant. Asterisks show significant between-drug differences. The effect of injection number on glucose response alone was not significant for both MDPV and cocaine (One-Way RM ANOVA). Overall locomotor responses during this time period showed no significant differences between drugs or injection numbers. Original cocaine data were previously reported in detail (Wakabayashi and Kiyatkin, 2015a) and shown here for comparison.

Next, we analyzed the NAc glucose and locomotor responses induced by each of the four repeated MDPV injections (Figure 2). While the initial injection of MDPV induced a modest down-up change in NAc glucose (Figure 2A), subsequent injections resulted in modest decreases in glucose for ~10 min post-injection (Figures 2B–D). However, total response assessed by AUC for 45 min post-injection was minimal with no significant differences between MDPV injections (Figure 2I). This stability of MDPV-induced glucose responses contrasted to progressive decreases in NAc glucose responses occurring during repeated cocaine injections (Figures 2A–D). However, locomotor responses remained relatively stable across repeated injections of both MDPV and cocaine, with no significant differences in amplitude, duration, and the AUC (Figures 2E–H,J).

**Figure 3:**
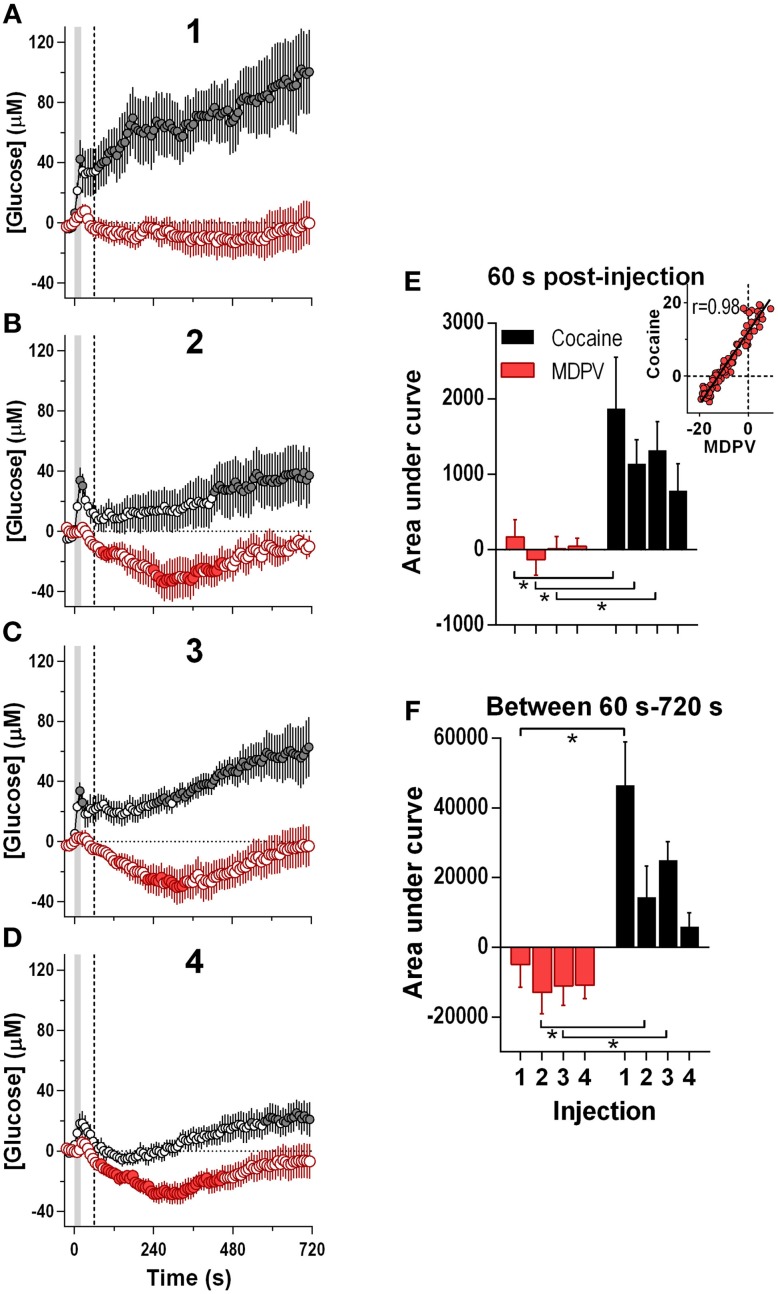
**Relative changes in NAc [glucose] induced by MDPV and cocaine injections (1–4) assessed at high temporal resolution (8-s bins)**. **(A–D)**, mean (±SEM) changes in NAc [glucose] (μM) induced by MDPV (red circles) and cocaine (gray circles) for 720 s after the injection onset. Vertical shaded area (starting at 0 s) marked the onset of 20-s MDPV injection. Horizontal dotted lines show basal levels (=0 μM). Concentration changes for the 720 s analysis were significant for 2–4 injections of MDPV [*F*_(7, 630)_ = 4.05, 3.73, 4.31, all *p* < 0.05] and 1–3 injections of cocaine [*F*_(6, 534)_ = 3.24, 1.83, 4.19, all *p* < 0.05]. Individual concentration values significantly different from baseline (Fisher test) are shown as filled symbols. **(E,F)**, mean ± SEM values of glucose responses induced by MDPV and cocaine as assessed by the area under the curve for 60 s post-injection **(E)** and between 60 s and 720 s post-injection **(F)**. Two-Way RM ANOVA analysis revealed a main effect of drug [*F*_(1, 39)_ = 13.9, *p* < 0.05] on NAc Glucose during 60 s post-injection. Neither Injection nor Interaction was significant. A similar analysis for the 60–720 s interval revealed a main effect of drug and injection [*F*_(1, 39)_ = 28.63, *F*_(3, 39)_ = 5.39, both *p* < 0.05). Interaction approached significance [*F*_(3, 39)_ = 2.73 *p* = 0.057]. Asterisks show significant between-drug differences. The effect of injection number alone was not significant for MDPV- or cocaine-induced [glucose] at this time interval (One-Way RM ANOVA). Inset in **(E)** shows the correlation between the mean [glucose] response for the first 180 s after cocaine and MDPV injections (*r* = 0.98). Original cocaine data were previously reported in detail (Wakabayashi and Kiyatkin, 2015a) and shown here for comparison.

Since the maximal between-drug differences in glucose dynamics were found within several minutes following injections, next our data were analyzed at a high time resolution (8-s bin) (Figure 3). In contrast to cocaine, which induced ultra-fast glucose rise within the first 60 s after each drug injection, MDPV within this time interval showed only weak, non-significant glucose increases (Figures 3A–E). These initial MDPV-induced increases were equally weak with each injection but the initial rises induced by cocaine decreased across repeated injections. However, MDPV induced a more prominent tonic decrease in NAc glucose within 60–720 s post-injection; this change remained relatively stable in amplitude, duration, and AUC (Figures 3A–D,F). Within this time window, cocaine induced greatest increases in NAc glucose during the first injection and this change rapidly became weaker such that the fourth response resembled that of MDPV. In this case, changes in glucose levels induced by both drugs tightly correlated (*r* = 0.97; see inset in Figure 3E).

### General pattern of temperature responses induced by MDPV and cocaine

**Figure 4:**
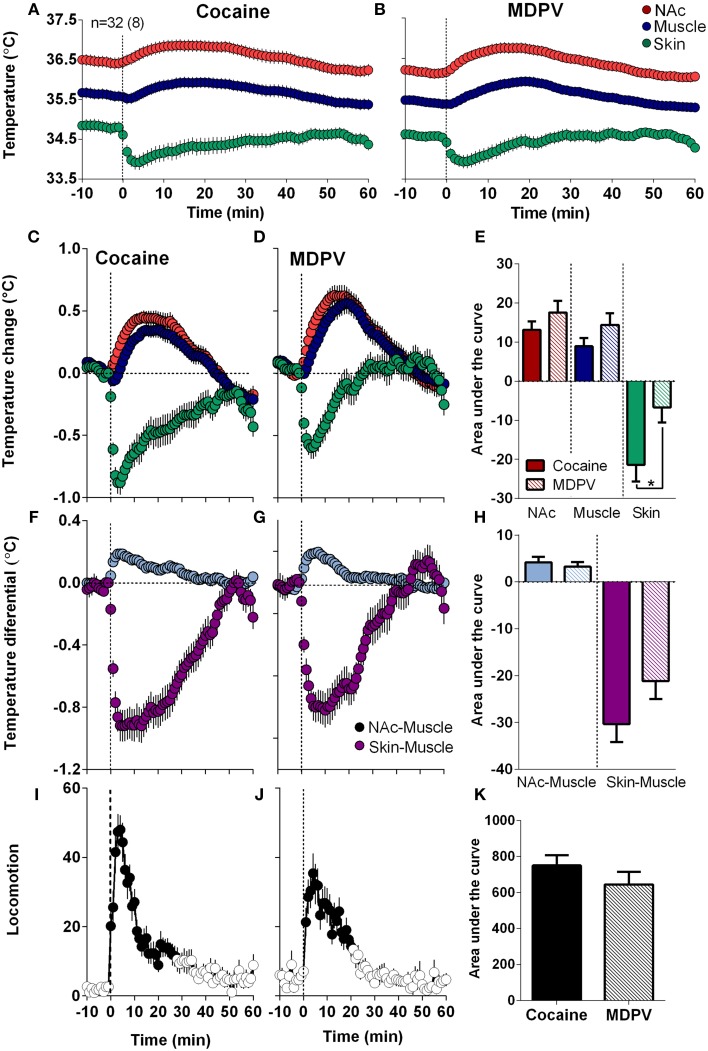
**Overall changes in temperature and locomotion induced by iv MDPV and cocaine**. **(A,B)**, mean ± SEM changes in absolute temperature recorded from the NAc, temporal muscle and skin. **(C,D)**, relative temperature changes in the same locations. **(F,G)**, changes in NAc-Muscle and Skin-Muscle temperature differentials. **(I,J)**, overall changes in locomotion. Data were obtained from eight rats (32 injections of each drug). Right panels show between-drug differences in the effects on brain temperature **(E)**, NAc-Muscle and Skin-Muscle differentials **(H)**, and locomotion **(K)** as assessed by the area under the curve for 45 min post-injection. Asterisk shows significant between drug differences for skin temperature (*p* < 0.05).

As shown in Figure 4, MDPV and cocaine induced similar temperature responses: NAc and muscle temperatures modestly increased for ~40 min, while skin temperature rapidly decreased, slowly returning to baseline at ~30–40 min (Figures 4A–D). Brain and muscle temperature increases evaluated by AUC for 45-min post-injection were slightly higher for MDPV than cocaine, but the difference was not significant. However, decreases in skin temperature induced by MDPV were significantly weaker than that for cocaine (Figure 4E).

Both drugs also similarly increased NAc-Muscle differentials and decreased Skin-Muscle differentials, suggesting metabolic brain activation coupled with skin vasoconstriction, respectively (Figures 4F,G). The increases in NAc-Muscle differentials were very similar for both drugs, but the decrease in Skin-Muscle differential for MDPV was weaker than that for cocaine; the difference approached statistical significance (*p* = 0.057) (Figure 4H). Both drugs also stimulated locomotion, with a sharper and shorter response for cocaine (Figures 4I,J), but the overall response was similar for both drugs (Figures 4I–K).

**Figure 5:**
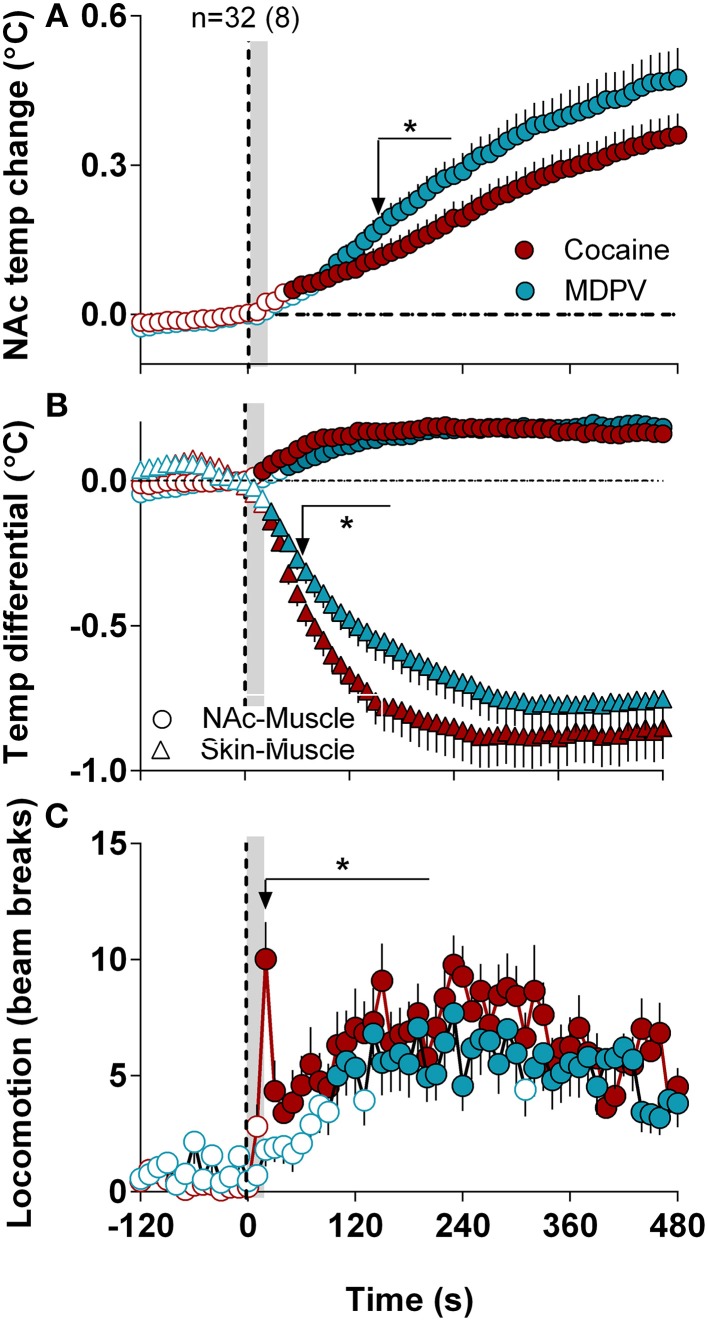
**High-resolution analysis of changes in temperature and locomotion induced by MDPV and cocaine**. **(A)**, mean ± SEM changes in NAc temperature. Two-Way RM ANOVA revealed a significant of Time [*F*_(49, 1519)_ = 80.05, *p* < 0.001] and Drug × Time Interaction [*F*_(49, 1519)_ = 4.12] with no effect of Drug [*F*_(1, 31)_ = 3.16, *p* = 0.08]. Small arrow with asterisk shows the moment (~160 s) when two curves significantly diverge from each other. **(B)** shows mean ± SEM changes in NAc-Muscle and Skin-Muscle differentials. Two-Way RM ANOVA revealed a main effect of Drug [*F*_(1, 31)_ = 4.32, *p* < 0.05], Time [*F*_(49, 1519)_ = 54.45, *p* < 0.01], and Drug × Time Interaction [*F*_(49, 1519)_ = 1.77, *p* < 0.01] on Skin-Muscle differential, with no significant changes for NAc-Muscle differential. **(C)**, mean ± SEM changes in locomotion. The between-drug difference for locomotion was significant only for 210 s after the injection onset [Drug × Time interaction *F*_(22, 682)_ = 1.57, *p* = 0.047]. Filled symbols in each graph show values significantly different from baseline revaluated by One-Way RM ANOVA.

When analyzed at a high time-resolution (10-s bins), MDPV displayed more rapid and stronger effects on NAc temperatures than cocaine [Time × Drug interaction *F*_(49, 1519)_ = 4.12, *p* < 0.05 (Figure 5A)]. Cocaine-induced NAc increase became significant at ~45 s, whereas MDPV-induced NAc increase became significant at 75 s from injection onset. The difference between curves became significantly different at ~140 s (see asterisk in Figure 5A). Both drugs had virtually identical effects on NAc-Muscle differentials, but the increase induced by cocaine had shorter rapid onset latency (15 vs. 45 s). Cocaine also displayed a significantly stronger effect on the Skin-Muscle differential [Drug *F*_(1, 31)_ = 4.32, Drug × Time interaction *F*_(49, 1519)_ = 1.77; both *p* < 0.05] (Figure 5B). The difference between curves became significant at ~50 s, and cocaine showed a much steeper, sharper drop in Skin-Muscle differential than MDPV.

Although both drugs induced locomotor activation, a noticeable difference was found during high-resolution analysis (Figure 5C). While MDPV-induced locomotor activation developed with a ~120-s latency, cocaine induced a sharp spike in motor activity during and immediately after the injection (~15 s).

### Temperature and locomotor effects of MDPV and cocaine following repeated drug injections

**Figure 6:**
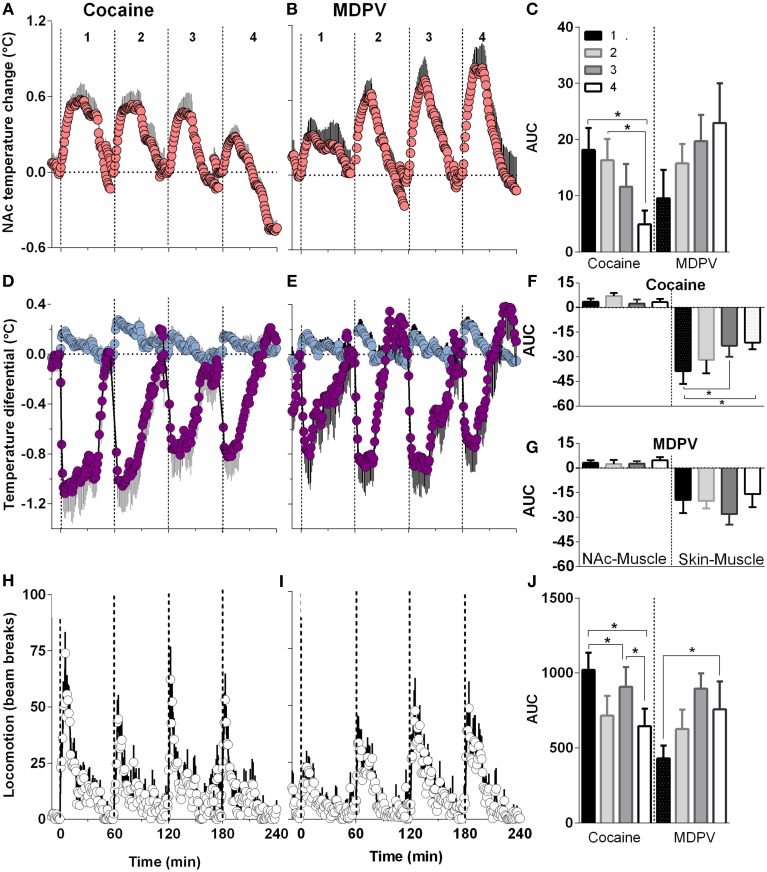
**Relative changes in NAc temperature (A,B), NAc-Muscle and Skin-Muscle differentials (D,E) and locomotion (H,I) induced by MDPV and cocaine injections (1–4)**. All parameters are shown with 1-min time resolution for 60 min post-injection. Right panels **(C,F,G,J)** shows differences in the effects of each drug depending upon injection number and evaluated as the area under the curve (AUC) for 45 min post-injection. For cocaine, One-Way RM ANOVA revealed significant effect on injection number on NAc temperature [*F*_(3, 21)_ = 6.25, *p* < 0.05], Skin-Muscle differential [*F*_(3, 21)_ = 4.07, *p* < 0.05] and locomotion [*F*_(3, 21)_ = 4.42, *p* < 0.05]. For MDPV, the effect was significant only for locomotion [*F*_(3, 21)_ = 3.15, *p* < 0.05].

When analyzed with respect to injection number, MDPV-induced increases in NAc temperature became larger with each subsequent injection (Figures 6B,C). This effect was evident with respect to response magnitude but the increase in the AUC did not reach statistical significance. Despite this apparent sensitization, both NAc-Muscle and Skin-Muscle differentials remained relatively constant for each subsequent injection (Figures 6E–G). A significant increase in locomotor activity [Figures 6I,J; *F*_(3, 21)_ = 3.15, *p* < 0.05] was also detected across injections.

Cocaine, on the other hand, showed a significant attenuation in NAc temperature responses across injections [*F*_(3, 21)_ = 6.25, *p* < 0.05; Figures 6A,C]. In addition, while NAc-Muscle differentials remained relatively constant, the decrease in Skin-Muscle differential attenuated over time [*F*_(3, 21)_ = 4.07, *p* < 0.05; Figures 6D,F,G]. Locomotor activity likewise significantly decreased during repeated cocaine injections [*F*_(3, 21)_ = 4.42, *p* < 0.05] (Figures 6H,J).

### The relationships between individual neurochemical and temperature parameters

**Figure 7:**
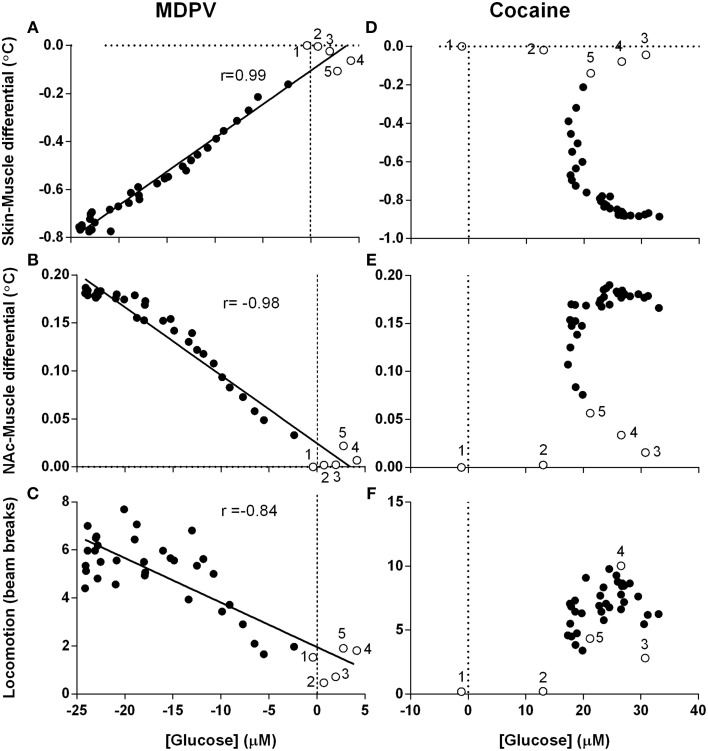
**Correlative relationships between changes in NAc glucose, temperature parameters and locomotion**. Pearson correlation coefficients and linear regression were calculated for all animals in all experimental sessions using mean, high-resolution (10-s bins) data and an analysis window of 360-s post-injection. Unfilled points labeled 1–5 represent the first five time points (-10 to 30 s) and correspond with effect latency. **(A–C)** show highly significant (*p* < 0.0001) and tight correlations between MDPV-induced changes in NAc [glucose] and **(A)** Skin-Muscle differential (*r* = 0.99, *y* = 0.028x - 0.104), **(B)** NAc-Muscle differential (*r* = −0.98, *y* = −0.007x + 0.025), and **(C)** locomotion (−0.84, *y* = −0.185x + 1.959). Data for cocaine is organized in parallel, with **(D–F)** displaying cocaine-induced NAc [glucose] changes vs. the same three parameters described above. *R*-values and linear regression are not shown for cocaine because of absence of correlation. Open circles with numbers are values for initial five time points (50 s) post-injection.

Since changes in [glucose] are determined by changes in metabolic activity and vessel tone, next we examined the relationships between drug-induced glucose responses, NAc-Muscle and Skin-Muscle differentials, two indices that reflect intra-brain heat accumulation due to metabolic activation and the state of skin vessels, respectively; the analysis window in this case was defined from the moment of drug injection (0 s) to the peak effect of MDPV on NAc glucose (360 s) (Figure 7).

When analyzed for MDPV, we found an exceptionally strong correlation between changes in glucose and Skin-Muscle differentials (Figure 7A; *r* = 0.99, *p* < 0.001), suggesting that the decrease in glucose could be related to drug-induced vasoconstriction. Additionally, as changes in Skin-Muscle differentials tightly correlate with changes in NAc-Muscle differentials (*r* = −0.99), MDPV-induced glucose decreases also showed a strong negative correlation with NAc-Muscle differentials (Figure 7B; *r* = −0.98). A slightly weaker, but also significant negative correlation was also found between glucose decrease and locomotor activity (Figure 7C; *r* = −0.84; *p* < 0.001).

When the same regression analysis was conducted for cocaine for the same time window, no clear correlation was apparent for all analyzed parameters. Both NAc-Muscle and Skin-Muscle differentials showed complex non-linear changes. For example, glucose levels strongly increased immediately after cocaine injection, but Skin-Muscle and NAc-Muscle differentials remained unaffected (see open circles for initial post-injection data points in Figures 7D,E). Locomotor activity similarly increased following a ~20-s delay, despite the rapid rise in NAc glucose (Figure 7F).

## Discussion

MDPV is a relatively new synthetic psychoactive drug of abuse and many issues regarding its mechanisms of action and physiological effects remain unknown. Based on its high affinity to the monoamine transporters and known similarity in stimulatory and sympathoexcitatory effects, MDPV is usually viewed as a more active cocaine-like drug. This study combined glucose electrochemistry, behavioral and temperature analyses to determine whether MDPV does in fact mimic the effects of cocaine and can accurately be described as “cocaine-like.” Here we show that iv MDPV and cocaine used at optimal self-administering doses similarly stimulate locomotion and moderately increase brain and body temperatures due to metabolic brain activation and peripheral vasoconstriction. Although there were minor between-drug differences in temperature and locomotor responses, MDPV and cocaine had startlingly opposite effects on NAc glucose, an important physiological parameter critical for the metabolic activity of brain cells.

### Mechanisms regulating brain glucose levels

Previous studies (Fellows et al., 1992; Silver and Erecinska, 1994; Attwell et al., 2010), including our recent work with electrochemical glucose monitoring (Kiyatkin and Lenoir, 2012; Kiyatkin and Wakabayashi, 2015) suggest that glucose entry into the brain extracellular space is governed by two different and simultaneously acting mechanisms. First, brain glucose levels could increase following robust increases in blood glucose levels. While such effects occur after high-dose glucose injections and consumption of high glucose-containing products (Wakabayashi and Kiyatkin, 2015b; Wakabayashi et al., 2015), blood glucose levels are tightly regulated and remained relatively stable under physiological conditions. Second, glucose entry could also be rapidly increased or decreased via local vasodilation or vasoconstriction that, respectively, enhances or diminishes local cerebral blood flow. This mechanism of active entry is neural activity-regulated, and thus is structure-specific (Attwell et al., 2010; Mergenthaler et al., 2013). For instance, in the NAc, arousing stimuli phasically excite most neurons and glucose levels rapidly rise. In the substantia nigra *pars reticulata*, however, most neurons are phasically inhibited by such stimuli and glucose levels are transiently lowered (Kiyatkin and Lenoir, 2012). Taking into account structural specificity, this mechanism allows for the redistribution of blood flow and thus delivery of oxygen and glucose to areas of high metabolic demand. Therefore, via neural regulation of local blood flow, the brain under physiological conditions is able to prevent any possible glucose deficit and anticipate future metabolic demands.

### Cocaine-like and cocaine-unlike effects of MDPV

If local vasodilation induced by neuronal activation is responsible for glucose entry into the extracellular space, it is reasonable to infer that constriction of the cerebral vessels will have the opposite effect. We found an exceptionally high correlation (*r* = 0.99) between MDPV-induced changes in NAc glucose and Skin-Muscle differential, a valid measure of vascular tone (Figure 7A). This suggests that constriction evident in the skin vessels also occurs in cerebral vessels within the NAc and may be responsible for the drug-induced decrease in glucose levels. Cocaine, however, induces a sharp rise in NAc glucose immediately after injection, despite inducing arguably stronger vasoconstriction. The next logical question that follows is: what is the cause of this sharp, unexpected difference?

Unlike MDPV, cocaine induces a very strong, but transient spike in motor activity immediately upon injection (see Figure 5C). This effect is distinct and masked in the slower, more tonic rise in locomotion that is similar for both MDPV and cocaine. Our previous studies in awake rats revealed that this ultra-fast motor effect of cocaine coincides with cortical EEG desynchronization, EMG activation (Kiyatkin and Smirnov, 2010), and phasic excitation of ventral striatal neurons (Kiyatkin and Brown, 2007). Therefore, the rapid rise in NAc glucose induced by iv cocaine could reflect local vasodilation and local increases in cerebral blood flow resulting from the activation of accumbal neurons, despite systemic vasoconstriction.

Therefore, the difference in glucose response between MDPV and cocaine could be primarily related to the unique properties of cocaine, particularly its ability to directly interact with ionic channels on afferents of sensory nerves (Lee et al., 2005) densely innervating blood vessels (Göder et al., 1993; Michaelis et al., 1994). Due to this action in the periphery and involvement of sensory pathways, iv delivered cocaine is able to induce phasic neural activation well before the drug reaches the brain and interact with centrally located neural substrates. This hypothesis was recently confirmed using cocaine-methiodide, a cocaine analog that cannot cross the blood-brain barrier (Shriver and Long, 1971; Hemby et al., 1994; Wise et al., 2008) but mimicked the initial rapid and strong effects of cocaine by engaging the same ionic channels in the afferents of peripheral sensory nerves. Similar to cocaine, cocaine-methiodide rapidly induced equally EEG desynchronization and EMG activation (Kiyatkin and Smirnov, 2010), phasically excited ventral striatal neurons (Kiyatkin and Brown, 2007), modestly increased brain and muscle temperatures (Kiyatkin and Brown, 2007), and lastly induced a virtually identical rapid glucose rise in the NAc (Wakabayashi and Kiyatkin, 2015a). Despite its inability to cross the blood-brain barrier, this drug induced skin vasoconstriction to levels comparable to cocaine, confirming previous findings on the central mediation of this peripheral effect (Knuepfer and Branch, 1992).

Although vasoconstriction induced by iv cocaine appears to be triggered primarily via its action on peripheral neural substrates (Knuepfer and Branch, 1992), the mechanisms underlying the slightly weaker, but still significant peripheral vasoconstriction induced by iv MDPV remain less clear. This effect is too rapid to reflect a direct action of this drug on centrally-located monoamine transporters. Possibly, MDPV interacts with peripherally located dopamine and norepinephrine transporters or has a direct, still undiscovered, direct action on adrenoceptors that controls vessel tone. Further experiments are necessary to clarify this issue.

In contrast to MDPV-induced glucose responses, which remained relatively stable with a tendency to increase across repeated injections, cocaine-induced glucose responses became weaker with each subsequent drug injection. MDPV-induced increases in NAc temperature and locomotion also remained relatively constant, while cocaine-induced increases in these parameters became weaker across injections. This apparent tolerance to cocaine is in contrast to the known sensitization of locomotor and temperature responses of cocaine seen with repeated day-to-day administration (Post et al., 1987; Kalivas et al., 1992; Blech-Hermoni and Kiyatkin, 2004), but is in concordance with the known tolerance of cardio-vascular effects of cocaine (Tella et al., 1999; Wilson et al., 2000). As such, sensory mechanisms involved in mediating the neural effects of cocaine may also hypothetically explain this difference. Similarly to cocaine, neural and temperature responses elicited by natural arousing stimuli also show progressive habituation following repeated exposure (Kiyatkin et al., 2002).

While local neural activation and local vasodilation (or increase in local cerebral blood flow) induced by cocaine is supported by multiple pieces of evidence obtained via different techniques (Stein and Fuller, 1993; Schmidt et al., 2006; Ceolin et al., 2007; Howell et al., 2010; Pan et al., 2014), other studies suggest that cocaine induces constriction of cerebral vessels and decreases cerebral blood flow (Du et al., 2009; Perles-Barbacaru et al., 2012). These results may differ due to brain structure specificity, varying drug doses, and routes of administration. However, in our view, the major factor in this discrepancy could be the use of general anesthesia, which is accompanied by peripheral vasodilation. In contrast to the awake state, the physiological effects of cocaine during anesthesia are drastically different at multiple levels, including motor and EEG activity, muscular tone, brain, body and skin temperatures, blood pressure response, etc… (Kiyatkin and Smirnov, 2010). While iv cocaine still desynchronized cortical activity when used during urethane anesthesia, this effect was dramatically inhibited and slowed (Kiyatkin and Smirnov, 2010), despite the known modest effects of this general anesthetic on transmission of sensory signals. Therefore, the neural effects of cocaine and local vascular and blood flow responses as seen in anesthetized animal preparations could be quite different from those recorded from the active brain of animals and humans.

### Functional implications

In contrast to MDPV, cocaine elicits a rapid peripherally triggered sensory signal, evidenced by the immediate post-injection surge in NAc glucose, the faster onset of temperature effects, and the initial, ultra-fast spike in motor activation. This interoceptive signal (i.e., sensory cue) induced by cocaine results in earlier and more direct reward signaling, which could be very important for the acquisition of drug-taking behaviors (see Wise and Kiyatkin, 2011 for review). As such, in the initial stages of recreational experimentation, cocaine users may be much more susceptible to a transition into addiction than MDPV-users. The presumed lack of primary sensory effects could also explain the high association of MDPV use with environmental (exteroceptive) stimuli. As MDPV is typically used by young adults under specific drug-taking environments such as parties (“raves”), intense sensory stimulation could act as conditioned, drug-associated environmental cues, which could be especially important during the transition from initial MDPV experimentation to chronic abuse. Although recent work suggests that rats readily acquire iv MDPV self-administration (Watterson et al., 2014; Aarde et al., 2015), the data regarding the development of MDPV self-administration vs. cocaine are absent and human evidence for intravenous MDPV-use is currently very limited.

Finally, similar to other psychomotor stimulants, MDPV has the potential to induce serious health complications, including robust hyperthermia and lethality (Ross et al., 2012; Kesha et al., 2013; Froberg et al., 2014). Although MDPV mimics the basic psychoactive effects of cocaine, it is at least 10-fold stronger and taking MDPV in place of cocaine may result in overdose or unexpectedly strong autonomic and psychoactive effects. Due to vasoconstriction coupled with local anesthetic action and sympathetic activation, cocaine appears to be more dangerous for the cardiovascular system than for the brain, where cocaine is able to induce neural activity-regulated vasodilation, thus increasing cerebral blood flow and delivery of glucose and oxygen to the brain. In contrast, MDPV may be more hazardous to the brain due to uncompensated cerebral vasoconstriction. In this respect, MDPV resembles METH, which has the potential to induce prolonged cerebral vasoconstriction and relative hypoxia that contributes to acute health complications following drug overdose (Polesskaya et al., 2011; Weaver et al., 2014).

## Author contributions

EK, KW, SR–concept and design of experiments; EK, KW, SR–data collection, assembly, data analyses and their interpretation; EK, KW, SR–drafting the manuscript. All authors approved the final version of the manuscript.

## Funding

These studies were supported by Intramural Research Program, NIH (1ZIADA000566-05, EK).

### Conflict of interest statement

The authors declare that the research was conducted in the absence of any commercial or financial relationships that could be construed as a potential conflict of interest.
